# The Impact of Age and Physical Assessment Measures on Performance Among Non-Weightbearing Walking Aids: A Randomized Clinical Trial

**DOI:** 10.1177/15563316261491728

**Published:** 2026-10-17

**Authors:** Audrey Zucker-Levin, Lucy Senger

**Affiliations:** 1 School of Rehabilitation Science, College of Medicine, University of Saskatchewan, Saskatoon, SK, Canada

**Keywords:** strength, perceived exertion, walking aid, age, plantar force

## Abstract

**Background:**

Walking aids for non-weightbearing (NWB) ambulation are often arbitrarily provided with no evidence that physical assessment predicts ambulatory performance.

**Purpose:**

We sought to determine if convenient physical assessment measures could guide NWB walking aid prescription and to determine how age impacts ambulatory performance.

**Methods:**

This prospective randomized crossover study examined 22 healthy adults, recruited from Saskatoon, Saskatchewan, and surrounding area through flyers and word of mouth. Inclusion criteria were independent unrestricted community dwellers with no self-reported neuromusculoskeletal or cardiovascular conditions who were age 18 years or older. Participants were stratified into 2 age cohorts: younger (age <50 years) and older (age ≥50 years). Physical assessment measures included triceps and latissimus dorsi strength, single lower-limb balance, and arm endurance. Performance measures included distance traversed, rate of perceived exertion, and peak stance limb plantar force (SLPF) evaluated among 4 walking conditions: unaided, NWB with crutches, standard walker, and wheeled knee walker. Two analyses were performed, one using the entire sample population and another split by age.

**Results:**

There were no differences in physical assessment measures between the younger and older cohort, yet distance traversed in the older cohort was highly dependent on all physical assessment measures when using crutches or a standard walker. Peak SLPF was reduced in the younger cohort when using the wheeled knee walker, but not in the older cohort. In the younger participants, reducing peak SLPF was highly dependent on arm endurance when using a standard walker or crutches and latissimus dorsi strength when using the wheeled knee walker. Surprisingly, older participants with stronger latissimus dorsi strength reported a higher rate of perceived exertion when using the wheeled knee walker compared to other devices.

**Conclusion:**

Arm strength, single lower-limb balance, and arm endurance should be considered when prescribing walking aids in older adults, and the wheeled knee walker should be considered in younger adults in need of reduced peak SLPF.

**Level of Evidence:**

Level III, prospective randomized comparative study.

## Introduction

Walking aid prescription for people in need of non-weightbearing (NWB) ambulation (full lower limb offloading) may be temporary after acute injury or surgical intervention or long term for chronic pathologies. Traditional devices used for NWB ambulation are axillary crutches and standard walkers. However, wheeled knee walkers (rolling knee scooters) have seen increased use, as they allow faster ambulation speed with less metabolic exertion and require less muscular recruitment.^1,2^ Other devices such as the hands-free single crutch and dynamic exoskeletal orthoses are available for NWB ambulation, but recent studies identify major limitations including increased balance and coordination demands with the hands-free single crutch when compared to forearm crutches^2^ and lower limb discomfort when using dynamic exoskeletal orthoses.^3^ Furthermore, use of walking aids is associated with a risk of falling. Nonetheless, there is no evidence of objective measures to guide the prescription of walking aids. Thus, prescription is often arbitrary based on availability, age, cost, and balance assessment, or they are acquired without professional guidance.^4,5^

Appropriate NWB walking aid selection requires clinicians to consider stability and mobility needs of the patient, while still protecting the weightbearing foot from excess force. Optimally, the walking aid would allow the user to safely continue with daily activities, limit sedentary behavior, minimize discomfort of the stance limb foot and arms, and dissuade walking aid rejection (morbidity of use) which is particularly common in older people in need of limb offloading.^1,6^

Walking aids are required over the aging continuum from childhood to older adults. Studies of walking aid use have reported on oxygen consumption, ambulation speed, and temporospatial kinetic and kinematic gait parameters with differences amongst devices. However, participants in these studies predominantly include younger, healthy adults.^3,7-9^ This is unfortunate as ankle fractures, a leading cause for NWB ambulation, occur most frequently in adolescents 10 to 19 years of age (23.5%) with adults 50 to 59 years of age, the next most prevalent group at 13.9% of the population.^10^

Research that has focused on walking aid use among older adults revealed that people chose the wheeled knee walker over crutches and standard walkers with demonstrated reduced stance limb plantar force (SLPF), greater ambulation speed and less perceived exertion when using the wheeled knee walker in comparison to crutches or a standard walker.^11^ Tangential work identified regional (rearfoot, midfoot, and forefoot) SLPF variance over time among walking aids with the greatest reduction in plantar force with wheeled knee walker use in adults over 50 years of age.^12^ Furthermore, significant reductions in hand loading and a 45% reduction of exertion were found when using a wheeled knee walker compared to crutches and standard walker in adults over 50 years of age with type 2 diabetes mellitus.^13^ This is important as crutch assisted ambulation transfers substantial body weight through the hands, wrists, and upper limbs, contributing to upper extremity strain and increasing the physical burden associated with use of these devices.^1,8^

Ambulatory performance measures are well described in the literature with a commonly held belief that stronger people with better balance and endurance accommodate to standard walkers and crutches better than their less fit counterparts. However, it is not clear which specific physical assessment measures impact NWB ambulation performance. The literature identifies the need to consider multiple variables including cognitive function, physical endurance, living environment, judgment, vision, vestibular function, and upper and lower body strength when prescribing walking aids, but there is no guideline or evidence of which physical assessment measures impact ambulatory performance.^1,14,15^

Identifying valid and reliable, readily available, time efficient physical assessment measures that can be performed in a variety of settings and do not require specialized equipment led the researchers to select 4 tests specific to needs of individuals using walking aids for NWB ambulation: standardized muscle strength testing of the triceps and latissimus dorsi^16,17^; the single limb balance test (also known as the single leg stance test, timed single limb stance, unipedal balance test, 1 leg stance test, and 1 leg standing balance test)^18^ and the seated push up test.^19^ Selection of these physical assessment measures was supported by prior evidence linking upper extremity strength, endurance, and postural control to mobility performance during assisted ambulation and aging-related functional decline.^1,14,20^ These tests focus on both strength and endurance of the muscles required during swing-through of the body and balance on the stance limb necessary when advancing the walking aid. Furthermore, all tests can be safely performed in the patient who requires NWB ambulation.

The ambulatory performance outcomes included distanced traversed, rate of perceived exertion, and peak SLPF, as elevated exertion or stance limb discomfort may limit walking tolerance, contribute to device abandonment, and promote sedentary behavior—particularly in older adults.^11,21^

Therefore, the objectives of this study were to determine if a subset of readily available and time efficient physical assessment measures could be identified to guide NWB walking aid prescription based on ambulatory performance in an indoor, level environment, and to determine how age impacts ambulatory performance with walking aids used for NWB.

The funders played no role in the design, conduct, or reporting of this study.

## Methods

Participants were recruited from Saskatoon, Saskatchewan, and surrounding area through flyers and word of mouth. Ethical approval was granted from the University of Saskatchewan (NER32), and informed consent was obtained prior to data collection. The study was registered on October 26, 2021, with ClinicalTrials.gov (NCT05092932)^11,22^; data from participants ≥50 years of age had been collected as part of the trial registered with ClinicalTrials.gov. (This study used the same University of Saskatchewan ethics approval (NER32) as the previously reported study,^11^ which allowed us to compare younger participants to the older participants.) Funding was provided by the University of Saskatchewan School of Rehabilitation Science student research grant; the funder had no role in design, conduct, data analysis, or reporting.

Inclusion criteria were age ≥18 years, independent unrestricted community dwellers with no self-reported neuromusculoskeletal or cardiovascular conditions. Individuals were excluded if they weighed more than 114 kg (weight limit of the wheeled knee walker) and/or had a history of walking aid use in the previous 5 years. All participants were asked to wear comfortable clothing and shoes they would normally wear for walking in a shopping center. Participants were informed they may choose to terminate the testing at any time if they so choose. Eligible participants attended a single testing session at the University of Saskatchewan.

A sample size of 22 participants was selected based on methodological precedence and statistical considerations for a within subjects experimental design.^7,11-13^ Participants answered basic demographic questions about age, sex, medical history, and history of walking aid use. Height and weight were recorded. Bilateral triceps and latissimus dorsi strength were each assessed 3 times using a handheld dynamometer (Commander Echo; JTech Medical) with the subject in the standard test position.^16,17^ Strength for each muscle was individually calculated as the average of the highest recorded measures for the right and left arms. Single limb balance was tested while the participant balanced on the foot randomly selected as the stance foot. A 60-second ceiling, which is slightly higher than the 45-second ceiling used in a published normative values study, was used because the published study reported their 18 to 39-year-old participants were able to achieve a mean of 45 seconds.^18^ Likewise, arm endurance was assessed with participants using push up stands (Kipika; Golimanor) to hold a bilateral press up in the seated position.^19^ A standard stopwatch (seconds) was used for both single limb balance and arm endurance measures with participants given 3 opportunities to achieve the 60-second ceiling with a 2-minute rest provided between trials. After physical assessment measures were obtained, participants were instructed on use of the Borg Revised Category-Ratio Scale-10 and were asked to rate perceived exertion, where 0 represented “nothing at all” and 10 represented “maximal” exertion.^17^ The weightbearing leg and walking aid test order were both randomized by a random number generator for each participant. Walking aids were identified (1-4) and the computer-generated random order was placed in unmarked envelopes drawn by participants. All participants were fit and instructed on the proper and safe use of 3 walking aids for NWB ambulation: axillary crutches (Guardian, Adult Quick-Fit; Medline Industries), standard walker (Duro-Med Folding Walker; Breg, Inc), and wheeled knee walker (Drive steerable aluminum knee walker; Medical Depot, Inc). All participants were provided a familiarization period with each device prior to data collection with testing performed after the participant indicated they felt safe walking with the walking aid.

A wireless insole force sensor (Loadsol, Novel Electronics, Inc) was fitted in the shoe of the stance foot to measure SLPF during each walking trial. The force sensor covered the entire plantar surface of the foot and was calibrated following the manufacturer’s protocol for each participant prior to data collection. Data were recorded at 100 Hz via Bluetooth connecting to an iOS device (iPad or iPhone).

A 2-minute walk test (2MWT) with the participant walking in a vacant 1.75-m wide, 60 m × 12 m rectangular hallway was then performed under the 4 conditions (unaided walking and for each walking aid). Participants were instructed to cover as much distance as safely possible in 2 minutes while a researcher followed with a standard measuring wheel (Uline) to record distance traversed. SLPF was recorded during the 2MWT and participants were asked to quantify their rate of perceived exertion after each walk. A mandatory 2-minute rest was provided after each walking trial; more rest was provided if requested.

Plantar forces were exported from the Loadsol application to Excel and visually screened for missing values or abnormal trends. The second 30 seconds epoch (from 30 to 60 seconds) of each walking trial was selected for analysis to allow participants sufficient time to reach steady-state walking under each condition. Peak SLPF was identified as the average highest total plantar force of all footfalls in the data window.

### Statistical Analysis

Statistical analyses were completed using Excel’s statistical toolbox (Microsoft). The cohort was examined as a whole and then stratified into 2 age cohorts: younger (<50 years) and older (≥50 years). Descriptive statistics were generated for all demographic data (age, sex, weight, and height), physical assessment measures (triceps and latissimus dorsi strength, single limb balance, and arm endurance), and ambulatory performance measures (distance traversed, rate of perceived exertion, and SLPF; Table 1).

**Table 1.** Mean (Standard Deviation) and Range of Participant Demographics, Physical Assessment Measures, and Performance Measures.

Variable/condition	Entire cohort (n = 22)	Older cohort: ≥50 y (n = 11)	Younger cohort: <50 y (n = 11)	Between-age cohort *T*-test comparison (≥50 vs <50 y)
Demographics				
Age (y), Range	46.2 (13.1), 23-60	56.3 (7.0), 50-60	36.2 (8.7), 23-47	0.00^a^
Male/female	6/16	3/8	3/8	-
Mass (kg), Range	74.6 (18.6), 53-120	70.9 (17.1), 53-111	81.8 (17.1), 59-120	0.10
Height (cm), Range	170.9 (9.5), 155-193	172.6 (8.9), 160-193	171.5 (8.8), 155-184	0.40
Physical assessment measures				
Triceps strength (N), Range	14.2 (5.7), 3.5-28.6	13.1 (6.0), 3.5-22.9	15.1 (5.3), 9.8-28.6	0.24
Latissimus dorsi strength (N), Range	20.4 (9.0), 7.7-40.7	19.39 (9.7), 7.7-40.7	21.3 (8.1), 13.0-36.1	0.32
Single limb balance (s), Range	55.6 (11.3), 20-60	51.5 (14.5), 20-60	60 (0)^b^, 60-60	0.10
Arm endurance (s), Range	38.5 (21.1), 1-60	36.6 (23.3), 1-60	40.5 (18.3), 8-60	0.35
Performance measures				
Distance traversed (M)				
Unaided	184.3 (37.5)^A^	193.3 (35.1)^A^	174.4 (37.5)^A^	0.14
Crutches	107.2 (41.3)	106.3 (42.8)	108.2 (39.5)	0.46
Walker	52.1 (20.6)	55.3 (23.8)	48.6 (15.6)	0.24
WKW	207.2 (55.3)^A^	215.8 (55.2)^A^	197.7 (53.9)^A^	0.24
RPE				
Unaided	0.9 (0.8)	1.2 (0.6)^B^	0.6 (0.8)	0.10
Crutches	5.8 (1.3)^A^	5.6 (1.4)^A^	5.9 (1.2)^A^	0.69
Walker	6.1 (1.4)^A^	5.9 (1.4)^A^	6.2 (1.3)^A^	0.64
WKW	2.7 (1.5)	2.4 (1.2)^B^	3.4 (1.3)	0.08
PSLPF (N)				
Unaided	1803.8 (613.7)^A^	1934 (655.0)^A^	1685.5 (547.2)^A^	0.38
Crutches	2197.3 (753.2)^A^	2322.7 (886.4)^A^	2071.8 (563.4)^A^	0.46
Walker	1992.7 (938.6)^A^	2290.0 (1076.0)^A^	1695.5 (653.7)^A^	0.15
WKW	1220.9 (525.6)	1369.1 (605.4)^A^	1072.7.0 (377.0)	0.20

Abbreviations: PSLPF, peak stance limb plantar force; RPE, rate of perceived exertion; Walker, standard walker; WKW, wheeled knee walker.

Values that share letters (A, B) within groups are NOT different at the *P* < .05 level.

a Indicates significant difference between young and older cohort *P* < .05.

b Indicates ceiling effect.

Independent samples *t*-tests were performed to confirm baseline comparability between age cohorts and to identify differences in physical assessment measures and ambulatory performance measures between the age cohorts. One-way repeated measures analysis of variance (ANOVA) was used to identify differences in distance traversed, rate of perceived exertion, and peak SLPF within cohorts and across walking aids. When significant differences were found, a Bonferroni correction was applied to conservatively adjust *P* values for multiple comparisons and identify pairwise differences. An a priori α = .05 was set for both *t*-test and ANOVA calculations. Finally, bivariate correlations were used to identify the association of clinical to performance measures with correlations identified as ≤0.39 weak, 0.40 to 0.59 moderate, 0.60 to 0.79 strong, and ≥0.80 very strong.^23^ This study and manuscript conform with CONSORT 2025 guidelines.

## Results

Twenty-two participants were tested and stratified evenly into younger (3 male, 36.2 ± 8.7 years, n = 11) and older (3 male, 56.3 ± 7.0 years, n = 11) age cohorts (Table 1). All participants were able to complete all testing conditions. No complaints or harms (pain, excess fatigue, and weakness) were identified.

No age group differences in baseline demographics, except for age (*P* = .00), were identified. Physical assessment measures and ambulatory performance measures did not differ between age cohorts (Table 1).

When the entire cohort was assessed, there were differences in distance traversed among walking aids (*P* < .001). Post-hoc tests identified differences (*P* < .001) between distance traversed among all walking conditions except for unaided to wheeled knee walker. Distance traversed unaided (184.3 ± 37.5 m) and with the wheeled knee walker (207.2 ± 55.3 m) were each greater than that traversed with crutches (107.2 ± 41.3 m) or a standard walker (52.1 ± 20.6 m); distance traversed with crutches was greater than that traversed with the standard walker.

Both the younger and older cohorts identified the same trend as the entire cohort. In the older cohort, distance traversed unaided (193.3 ± 35.1 m) and with the wheeled knee walker (215.8 ± 55.2 m) were each greater than that traversed with crutches (106.3 ± 42.8 m) or a standard walker (55.3 ± 23.8 m); likewise, in the younger cohort, distance traversed unaided (174.4 ± 37.5 m) and with the wheeled knee walker (197.7 ± 53.9 m) were each greater than that traversed with crutches (108.2 ± 39.5 m) or a standard walker (48.6 ± 15.6 m). In both age cohorts, distance traversed with crutches was greater than that traversed with the standard walker.

Distance traversed strongly correlated with triceps (*r* *=* 0.60) and latissimus dorsi (*r* *=* 0.61) strength in the entire cohort when using the standard walker and with arm endurance when using crutches (*r* *=* 0.64). Age-specific analyses showed in the older cohort all physical assessment measures strongly correlated with distance traversed when using the standard walker (triceps strength *r* *=* 0.71, latissimus dorsi strength *r* *=* 0.70, single leg balance = 0.71, and arm endurance *r* *=* 0.71) and all but latissimus dorsi strength strongly correlated with distance traversed when using crutches (triceps strength *r* *=* 0.72, single lower limb balance *r* *=* 0.79, and arm endurance *r* *=* 0.71). When physical assessment measures were correlated with distance traversed, no strong correlations were identified in the younger cohort. A ceiling effect prevented calculation of single lower limb balance as all participants were able to balance for ≥60 seconds (Table 2).

**Table 2.** Pearson Correlation Coefficient *r* Values for the Relationships Between Physical Assessment Measures (Triceps Strength, Latissimus Dorsi Strength, Single Limb Balance, and Arm Endurance) and Performance Measures (Walking Distance, Rate of Perceived Exertion, and Peak Stance Limb Plantar Force) for the Entire Cohort (n = 22) and for the Younger and Older Cohorts (n = 11 Each) Among Walking Aids.

Variable/condition	Triceps strength	Latissimus dorsi strength	Single limb balance	Arm endurance
Distance traversed				
Entire cohort (n = 22)				
Unaided walking	−0.11^a^	0.04^a^	0.08^a^	0.20^a^
Crutches	0.47^b^	0.30^a^	0.56^b^	**0.64** ^c^
Standard walker	**0.60** ^c^	**0.61** ^c^	0.40^b^	0.40^b^
Wheeled knee walker	0.24^a^	0.49^b^	0.27^a^	0.35^a^
Older cohort (n = 11)				
Unaided walking	0.11^a^	0.39^a^	0.28^a^	0.36^a^
Crutches	**0.72** ^d^	0.37^a^	**0.79** ^d^	**0.79** ^d^
Standard walker	**0.71** ^d^	**0.70** ^d^	**0.71** ^d^	**0.71** ^d^
Wheeled knee walker	0.39^a^	0.55^b^	0.39^a^	0.50^b^
Younger cohort (n = 11)				
Unaided walking	−0.27^a^	−0.30^a^	CE	0.06^a^
Crutches	0.13^a^	0.21^a^	CE	0.44^b^
Standard walker	0.57^b^	0.55^b^	CE	0.19^a^
Wheeled knee walker	0.15^a^	0.42^b^	CE	0.52^b^
Rate of perceived exertion				
Entire cohort (n = 22)				
Unaided walking	−0.32^a^	−0.23^a^	−0.28^a^	−0.23^a^
Crutches	−0.14^a^	−0.07^a^	0.01^a^	0.52^b^
Standard walker	0.02^a^	−0.07^a^	0.26^b^	0.48^b^
Wheeled knee walker	0.29^b^	0.46^b^	−0.04^a^	0.06^a^
Older cohort (n = 11)				
Unaided walking	−0.41^b^	−0.33^b^	−0.25^a^	−0.14^a^
Crutches	−0.09^a^	0.18^a^	−0.03^a^	−0.13^a^
Standard walker	−0.16^a^	−0.28^a^	0.31^b^	−0.17^a^
Wheeled knee walker	0.30^a^	**0.69** ^c^	−0.30^b^	−0.03^a^
Younger cohort (n = 11)				
Unaided walking	−0.11^a^	−0.11^a^	CE	−0.57^b^
Crutches	−0.38^a^	−0.57^b^	CE	0.43^b^
Standard walker	0.50^b^	0.31^a^	CE	−0.19^a^
Wheeled knee walker	0.25^a^	0.19^a^	CE	0.16^a^
Peak stance limb plantar force				
Entire cohort (n = 22)				
Unaided walking	−0.33^a^	−0.32^a^	0.02^a^	−0.29^a^
Crutches	−0.01^a^	0.07^a^	0.16^a^	−0.06^a^
Standard walker	0.13^a^	0.02^a^	0.13^a^	−0.19^a^
Wheeled knee walker	−0.18^a^	−0.07^a^	0.03^a^	−0.14^a^
Older cohort (n = 11)				
Unaided walking	−0.16^a^	0.00^a^	0.12^a^	−0.09^a^
Crutches	0.16^a^	0.40^b^	0.26^a^	0.19^a^
Standard walker	0.46^b^	0.35^a^	0.30^a^	0.08^a^
Wheeled knee walker	0.07^a^	0.25^a^	0.16^a^	−0.08^a^
Younger cohort (n = 11)				
Unaided walking	−0.48^b^	**−0.72** ^c^	CE	**−0.60** ^c^
Crutches	−0.28^a^	−0.53^b^	CE	**−0.60** ^c^
Standard walker	−0.38^a^	−0.59^b^	CE	**−0.80** ^d^
Wheeled knee walker	−0.55^b^	**−0.64** ^c^	CE	−0.21^a^

Abbreviation: CE, ceiling effect. Bolded text indicates strong or very strong correlations.

a Weak correlation (≤0.39).

b Moderate correlation (0.40-0.59).

c Strong correlation (0.60-0.79).

d Very strong correlation (≥0.80).

When the cohort was assessed as a whole, ANOVA identified differences in rate of perceived exertion among walking aids (*P* < .001). Post-hoc tests identified significant differences (*P* < .001) in rate of perceived exertion among all walking conditions except for standard walker to crutches. Rate of perceived exertion unaided (0.9 ± 0.8) was less than with the wheeled knee walker (2.7 ± 1.5), crutches (5.8 ± 1.3), or a standard walker (6.1 ± 1.4); rate of perceived exertion and wheeled knee walker were less than crutches and standard walker (Table 1).

Differences among walking aids were identified in both the younger and older cohorts when each were separately examined (*P* < .001). The pattern in the younger cohort was the same as the entire cohort with significant differences (*P* < .001) in rate of perceived exertion among all walking conditions except for standard walker to crutches. Rate of perceived exertion unaided (0.6 ± 0.8) was less than with the wheeled knee walker (3.4 ± 1.3), crutches (5.9 ± 1.2), or a standard walker (6.2 ± 1.3); rate of perceived exertion with wheeled knee walker was less than crutches and standard walker. The pattern differed in the older cohort with rate of perceived exertion unaided (1.2 ± 0.6) and with the wheeled knee walker (2.4 ± 1.2) each less than with crutches (5.6 ± 1.4) or standard walker (5.9 ± 1.4) but rate of perceived exertion was not different when unaided and wheeled knee walker was compared, likewise rate of perceived exertion did not differ when comparing standard walker and crutches (Table 1). The only strong correlation of rate of perceived exertion with physical assessment measures was in the older cohort with latissimus dorsi strength strongly correlating with rate of perceived exertion when using the wheeled knee walker (*r* *=* 0.69; Table 2).

When the entire cohort was examined, a single factor ANOVA identified differences in peak SLPF among walking aids (*P* < .001). Post-hoc tests identified significant differences (*P* < .001) in peak SLPF between the wheeled knee walker (1220.0 ± 525.6 N) and all other walking conditions: unaided (1803.8 ± 613.7 N), crutches (2197.3 ± 753.2 N), and standard walker (1220.9 ± 525.6 N). No other differences were identified (Table 1). When the younger cohort was examined, significant differences in peak SLPF among walking aids were identified (*P* < .001). Post-hoc analysis revealed stance limb peak force was lower with wheeled knee walker (1072.7 ± 377.0 N) than when walking unaided (1685.5 ± 547.2 N), or when walking with crutches (2071.8 ± 563.4 N). No other differences were identified. When the older cohort was examined, no differences in peak SLPF were identified among walking conditions.

No strong correlations were identified when physical assessment measures were correlated with SLPF in the entire cohort or in the older cohort. In the younger cohort peak SLPF strongly negatively correlated with latissimus dorsi strength when walking unaided (*r* *=* −0.72) and when walking with the wheeled knee walker (*r* *=* −0.64). Strong negative correlations were also found with arm endurance and peak SLPF when walking unaided (*r* *=* −0.60) and when walking with crutches (*r* *=* −0.60). Peak SLPF when walking with a standard walker very strongly negatively correlated with arm endurance (*r* *=* −0.80) in the younger cohort (Table 2).

## Discussion

This study sought to identify convenient physical assessment measures that could guide the prescription of walking aids for NWB ambulation and to determine whether age influences ambulatory performance and reliance on these measures in an indoor, level environment. Although assistive device selection is known to affect safety, exertion, and functional mobility during NWB ambulation, clinically feasible measures capable of informing device prescription remain poorly defined. To address this gap, we examined a cohort of healthy adults and stratified participants by age (<50 and ≥50 years) to determine whether convenient physical assessment measures, including triceps and latissimus dorsi strength, single lower limb balance, and arm endurance were associated with performance outcomes (distance traversed, rate of physical exertion, and peak SLPF) across different walking aids. We found that distance traversed in the older cohort was highly dependent on all physical assessment measures when using crutches or a standard walker. Peak SLPF was reduced in the younger cohort when using the wheeled knee walker but not in the older cohort. In the younger participants, reducing peak SLPF was highly dependent on arm endurance when using a standard walker or crutches and latissimus dorsi strength when using the wheeled knee walker. Surprisingly, older participants with stronger latissimus dorsi strength reported a higher rate of perceived exertion when using the wheeled knee walker compared to other devices.

This study has limitations. The sample size, although based on methodological precedence, was small. The ceiling effect of single limb balance in the younger cohort may have limited findings.

Age was the only measure that differed between age cohorts among baseline or physical assessment measures providing a stable base for between group comparison. When the cohort was analyzed as a whole, walking distance was greatest during unaided ambulation and when using the wheeled knee walker, with both conditions exceeding performance with crutches or a standard walker. This pattern persisted across age cohorts and is consistent with previous studies demonstrating greater walking distance and efficiency with wheeled knee walker use compared to traditional crutches or standard walkers.^6-8,12,24^ The absence of age-related differences in distance traversed suggests that healthy older adults can achieve comparable short-term mobility performance to younger adults when appropriate devices are used.

However, correlation of clinical to performance measures revealed important age-dependent differences. In participants age ≥50 years, distance traversed when using crutches or a standard walker strongly correlated with triceps strength, arm endurance, and single lower limb balance, while latissimus dorsi strength additionally showed a strong correlation with walking distance when using a standard walker. In contrast, strong correlations were absent in younger participants. These findings suggest that older adults rely more heavily on upper extremity strength, endurance, and postural control to compensate for the biomechanical demands of NWB ambulation with crutches or standard walkers.

Perceived exertion was consistently greatest when participants used a standard walker or crutches and lowest during unaided ambulation, with wheeled knee walker use producing intermediate rate of perceived exertion values. These results are aligned with other reports of higher metabolic demand and subjective effort during crutch and standard walker use compared with wheeled or hands-free mobility aids.^3,7,11,13^ Interestingly, latissimus dorsi strength highly correlated with rate of perceived exertion in the older cohort when walking with the wheeled knee walker. This was an unexpected finding that may be related to the need to stabilize the trunk more when using the wheeled knee walker to accommodate the faster ambulation speed (as identified by greater distance traversed in the 2MWT). Alternatively, it may be related to a fear of falling, as the wheeled knee walker continues to roll when the stance limb is pushing in stance phase and when off the floor in swing phase. Older participants did need more time to become comfortable using the wheeled knee walker than the younger participants with hesitation voiced by some. However, fitting the device and practicing in a safe environment with a trained person did facilitate confidence in their abilities. This may not always be the case, as other investigators reported a very high rate of falling (up to 43%) in participants using a wheeled knee walker after foot and ankle surgery.^5,25^ This high rate of falling may have occurred because the patients did not receive training or education on use of the wheeled knee walker or they may have been traversing on rugged terrain or going downhill.^5^

We found novel age-specific peak SLPF patterns among walking aids in our participants. In both the entire cohort and the younger cohort, peak SLPF diminished when using the wheeled knee walker and increased when using crutches or a walker when compared to unaided walking. These findings are supported by previous comparison reports.^11,12^ The wheeled knee walker may increase body weight support as weightbearing through the lower extremities is more comfortable than through the upper extremities as done with a standard walker or crutches.^13^ However, in contrast to published reports, peak SLPF did not differ among walking aids in the older cohort, eliminating the protective effect of wheeled knee walker use in adults aged ≥50 years. Furthermore, peak SLPF did not correlate strongly with any physical assessment measures in the older participants, indicating that increased strength or balance capacity may not mitigate stance-limb loading in this age group. Conversely, in younger adults, greater arm endurance correlated very strongly with decreased peak SLPF when walking with a standard walker and strongly correlated with decreased peak SLPF when walking with crutches or when walking unaided. Likewise, latissimus dorsi strength strongly correlated with decreased peak SLPF when using the wheeled knee walker or when walking unaided.

Although physical assessment measures did not differ among the age cohorts, ambulatory performance was drastically different specifically in the ability to decrease peak SLPF when using walking aids. Thus, these findings underscore the importance of incorporating objective physical assessment measures into walking aid prescription, particularly for adults age 50 years old and older. Upper extremity strength, arm endurance, and single lower limb balance appear especially relevant when considering crutches or walkers in this population, whereas these factors may be less critical for younger adults with similar strength and arm endurance. The wheeled knee walker consistently supported greater walking distance with lower perceived exertion, supporting its consideration as a preferred option for NWB ambulation when feasible. However, reduced stance-limb loading with wheeled knee walker use cannot be assumed in older adults, emphasizing the need for individualized assessment.

At present, no formal guidelines based on convenient physical assessment measures exist to guide walking aid prescription for NWB ambulation. These results contribute evidence toward an objective, patient-specific framework for device selection, with the potential to optimize mobility, reduce exertional burden, and improve adherence during rehabilitation.

These findings suggest further exploration is needed into valid and reliable, readily available, and time-efficient physical assessment measures on performance in patient populations and in the real-world walking environment, ultimately to aid in the creation of guidelines for prescribing walking aids for NWB ambulation.

## Supplemental Material

sj-docx-1-hss-10.1177_15563316261491728 – Supplemental material for The Impact of Age and Physical Assessment Measures on Performance Among Non-Weightbearing Walking Aids: A Randomized Clinical Trial Supplemental material, sj-docx-1-hss-10.1177_15563316261491728 for The Impact of Age and Physical Assessment Measures on Performance Among Non-Weightbearing Walking Aids: A Randomized Clinical Trial by Audrey Zucker-Levin and Lucy Senger in HSS Journal®

sj-docx-2-hss-10.1177_15563316261491728 – Supplemental material for The Impact of Age and Physical Assessment Measures on Performance Among Non-Weightbearing Walking Aids: A Randomized Clinical Trial Supplemental material, sj-docx-2-hss-10.1177_15563316261491728 for The Impact of Age and Physical Assessment Measures on Performance Among Non-Weightbearing Walking Aids: A Randomized Clinical Trial by Audrey Zucker-Levin and Lucy Senger in HSS Journal®
